# The effect of pre-exercise alkalosis on lactate/pH regulation and mitochondrial respiration following sprint-interval exercise in humans

**DOI:** 10.3389/fphys.2023.1073407

**Published:** 2023-01-27

**Authors:** Claire Thomas, Rémi Delfour‐Peyrethon, Karen Lambert, Cesare Granata, Thomas Hobbs, Christine Hanon, David J. Bishop

**Affiliations:** ^1^ LBEPS, Univ Evry, IRBA, University Paris Saclay, Evry, France; ^2^ French Institute of Sport (INSEP), Research Department, Laboratory Sport, Expertise, and Performance, Paris, France; ^3^ Institute for Health and Sport (iHeS), Victoria University, Melbourne, VIC, Australia; ^4^ PhyMedExp, University of Montpellier, INSERM U1046, CNRS UMR 9214, Montpellier, France; ^5^ Department of Diabetes, Central Clinical School, Monash University, Melbourne, VIC, Australia; ^6^ Institute for Clinical Diabetology, German Diabetes Center, Leibniz Center for Diabetes Research at Heinrich Heine University Düsseldorf, Düsseldorf, Germany; ^7^ German Center for Diabetes Research, Partner Düsseldorf, München-Neuherberg, Germany; ^8^ French Athletics Federation, Paris, France

**Keywords:** lactate, sodium bicarbonate, mct1, metabolic acidosis, lactate transport, mitochondrial function, MCT4, NBC

## Abstract

**Purpose:** The purpose of this study was to evaluate the effect of pre-exercise alkalosis, induced *via* ingestion of sodium bicarbonate, on changes to lactate/pH regulatory proteins and mitochondrial function induced by a sprint-interval exercise session in humans.

**Methods:** On two occasions separated by 1 week, eight active men performed a 3 × 30-s all-out cycling test, interspersed with 20 min of recovery, following either placebo (PLA) or sodium bicarbonate (BIC) ingestion.

**Results:** Blood bicarbonate and pH were elevated at all time points after ingestion in BIC vs PLA (*p* < 0.05). The protein content of monocarboxylate transporter 1 (MCT1) and basigin (CD147), at 6 h and 24 h post-exercise, and sodium/hydrogen exchanger 1 (NHE1) 24 h post-exercise, were significantly greater in BIC compared to PLA (*p* < 0.05), whereas monocarboxylate transporter 4 (MCT4), sodium/bicarbonate cotransporter (NBC), and carbonic anhydrase isoform II (CAII) content was unchanged. These increases in protein content in BIC vs. PLA after acute sprint-interval exercise may be associated with altered physiological responses to exercise, such as the higher blood pH and bicarbonate concentration values, and lower exercise-induced oxidative stress observed during recovery (*p* < 0.05). Additionally, mitochondrial respiration decreased after 24 h of recovery in the BIC condition only, with no changes in oxidative protein content in either condition.

**Conclusion:** These data demonstrate that metabolic alkalosis induces post-exercise increases in several lactate/pH regulatory proteins, and reveal an unexpected role for acidosis in mitigating the loss of mitochondrial respiration caused by exercise in the short term.

## 1 Introduction

During high-intensity exercise, the rapid increase in the energy demand of contracting skeletal muscles is associated with an increased rate of glycolysis and the subsequent accumulation of lactate anions and protons. Muscle lactate and proton accumulation are regulated by two lactate/proton cotransporter isoforms expressed in mammalian skeletal muscle, monocarboxylate transporter 1 (MCT1) and 4 (MCT4) (Brooks, 2018). Lactate is predominantly consumed by oxidative skeletal muscle fibres, after conversion to pyruvate and subsequent oxidation in the mitochondria. With different Michaelis–Menten constants (indicative of different binding affinities for lactate) (Dimmer et al., 2000), and evidence MCT1 is more abundant in type I muscle fibres (Pilegaard et al., 1999), while MCT4 is more abundant in type II fibres (Pilegaard et al., 1999; Dimmer et al., 2000; Hashimoto et al., 2005), it is thought the MCT isoforms may have different roles in different fibre types in human skeletal muscle.

Although lactate transporters provide the largest portion of muscle H+ removal during high-intensity exercise (Juel, 1996; Manning Fox et al., 2000), other sarcolemmal proteins are known to enhance both the transport activity of MCT1 and muscle pH regulation (Bangsbo et al., 1993; 1997; Becker et al., 2004; 2011; Becker and Deitmer, 2008). The MCTs need the action of their chaperone basigin (also referred to as CD147) to be correctly targeted to the membrane (Kirk et al., 2000). In addition, both the Na+/bicarbonate cotransporter (NBC) (Becker et al., 2004) and carbonic anhydrase isoform II (CAII) (Becker and Deitmer, 2008; Becker et al., 2011) have been reported to enhance the transport activity of MCT1. An estimated one-third of H+ removal during high-intensity exercise is non-lactate coupled (Bangsbo et al., 1993; 1997), primarily through the sodium/hydrogen exchanger (NHE) and NBCs.

The effects of a single session of high-intensity exercise on skeletal muscle are still equivocal. Some studies have shown positive changes to molecular processes involved in metabolism after high-intensity exercise, including increased MCT protein content (Coles et al., 2004; Bickham et al., 2006; Bishop et al., 2007), as well as stimulation of mitochondrial biogenesis and improvements to mitochondrial morphology (Little et al., 2011; Kitaoka et al., 2012; Ikeda et al., 2013). Other studies have reported contrasting results, with significant decreases in lactate transporter content (Tonouchi et al., 2002; Bishop et al., 2007) and activity (Dubouchaud et al., 1999; Eydoux et al., 2000), muscle buffer capacity (Bishop et al., 2009), and muscle oxidative capacity (Gollnick et al., 1990; Fernström et al., 2007; Larsen et al., 2016; Layec et al., 2018) after exercise performed until exhaustion. These findings suggest that impairment in the lactate shuttle, in proteins involved in muscle pH regulation, and muscle oxidative capacity, may occur during the recovery from high-intensity exercise. However, further research is required to confirm these findings and to investigate the time course of any changes.

While lactate is now well-recognised as an important signaling molecule that mediates exercise-induced adaptations (Hashimoto et al., 2007; 2013; Latham et al., 2012; Porporato et al., 2012; Hoshino et al., 2015), there is also evidence that proton accumulation can have a negative effect on skeletal muscle adaptations (Edge et al., 2015; Genders et al., 2019). Indeed, metabolic acidosis has been reported to inhibit skeletal muscle oxidative capacity (Jubrias et al., 2003; Genders et al., 2019), and increase muscle protein breakdown (Mutsvangwa et al., 2004). These findings may help to explain previous reports that bicarbonate ingestion prior to high-intensity exercise training sessions is associated with greater training-induced changes in lactate transporters (Thomas et al., 2007), maximal muscle oxidative capacity (Bishop et al., 2010), and anaerobic performance (Wang et al., 2019).

Given that sodium bicarbonate ingestion can reduce exercise-induced metabolic acidosis (Hollidge-Horvat et al., 2000; Bishop et al., 2004), we hypothesised that ingestion of sodium bicarbonate may also affect acute exercise-induced changes in lactate/pH transport proteins and mitochondrial respiratory function. As we have reported that repeated sprints interspersed with long rest periods induced large metabolic perturbations in world-class athletes (Thomas et al., 2021), we, therefore, investigated the effects of pre-exercise alkalosis on changes in the content of MCT1 and MCT4, and other proteins implicated in muscle pH regulation, as well as on mitochondrial respiration, during the 24 h following this type of all-out sprint-interval exercise interspersed with long recovery periods.

## 2 Material and methods

### 2.1 Participants and ethical approval

The study design was approved by the Human Research Ethics Committee of Victoria University, Melbourne, Australia, and was carried out in accordance with the Declaration of Helsinki. A power analysis was performed using exercise-induced changes in the Blood bicarbonate concentration with BIC supplementation and in the protein content of MCT1 from preliminary data from our group. Based on these results and the ability to detect a change of at least 20% with a significance of 0.05 and a power of 0.8, the required number of participants was 7. To account for drop-outs or unforeseen circumstances we aimed to recruit 25% more participants than needed; hence, the sample size was increased to *n* = 9. This sample size is consistent with many studies investigating the effects of exercise in humans (Little et al., 2011; Thomas et al., 2021).

Nine healthy active men, who performed physical activity 1 to 3 times per week, volunteered to participate in this study. We opted to recruit only a single-sex to keep the group of participants as homogeneous as possible, as previous research shows sex differences in response to exercise training in, for example, skeletal muscle substrate metabolism and the underpinning molecular mechanisms, mitochondrial ultrastructure (Lundsgaard and Kiens 2014). All participants were deemed healthy based on their responses to a medical screening questionnaire and were informed about the risks and requirements of the experiment before giving written informed consent. One participant was subsequently removed as a precaution, due to an adverse reaction to the initial muscle biopsy procedure. Descriptive characteristics for the eight participants who completed the study are as follows (mean ± SD): age 22 ± 4 years, height 179.6 ± 5.7 cm, mass 80.3 ± 13.0 kg, maximal oxygen uptake (VO_2max_) 44.3 ± 5.5 mL∙min^−1^∙kg^−1^.

### 2.2 Experimental design

**FIGURE 1:**
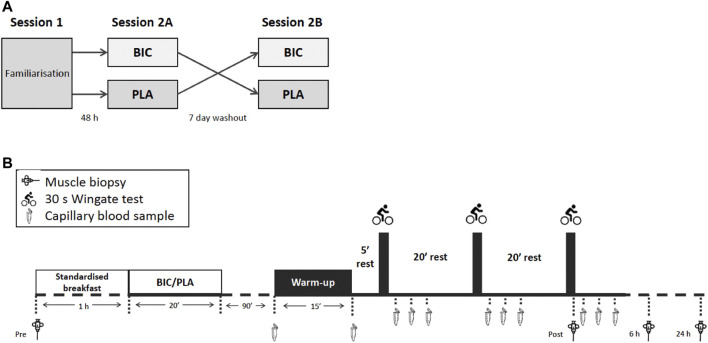
**(A)** An overview of the crossover experimental design, and **(B)** an overview of the experimental sessions 2A and 2B.

An overview of the experimental design is given in Figure 1A. Participants visited the laboratory for initial testing and a familiarisation session (Session 1), during which anthropometric measures were taken. The session also included a graded exercise test (GXT), a force-velocity test (FVT), and a single 30-s Wingate test which served as a familiarisation for the supramaximal exercise test in later sessions. The end of the GXT and the start of the FVT were separated by at least 5 h. A minimum of 48 h after completing the FVT, participants returned to the laboratory for the first sprint-interval Wingate session (Session 2A). After an overnight fast, participants had a resting muscle biopsy, and then a standardised breakfast routinely used in our lab [consisting of one serving of Sustagen^®^ (15 g diluted in 150 mL of water = 11 g of carbohydrate, 2.9 g of protein, 0.2 g of fat) + 1 banana], followed by either sodium bicarbonate or placebo supplementation. 90 min after the supplementation, participants then performed 3 × 30-s Wingate tests, each separated by 20 min of rest after the first and second all-out exercises. Immediately after the last Wingate test and 6 h later, skeletal muscle biopsies were collected. Twenty-four hours after the end of the exercise, participants returned to the laboratory for another biopsy after an overnight fast. All participants returned 7 days later (the same day and same hour as the first resting biopsy) to perform an identical testing session procedure with the alternative supplement (Session 2B). All exercise tests were performed in a laboratory where temperatures ranged between 20°C and 22°C. Strong verbal encouragement was provided to each participant during all maximal exercises. To minimise any potential exercise and/or diet-induced variability in measurements, participants were asked to refrain from vigorous activity and the ingestion of caffeine, alcohol, or other drugs for 48 h before and during sessions 2A and 2B, They could drink water *ad libitum*, but they were asked to consume exactly the same quantities of water from one condition to the next. Energy or isotonic drinks were also prohibited so as not to interfere with the supplementation and thus impact the subsequent biological analyses during the experiment. In this line, they received standardised breakfast and lunch.

#### 2.2.1 Session 1

##### 2.2.1.1 Graded Exercise Test (GXT)

All participants performed a graded exercise test on an electromagnetically-braked cycle ergometer (Lode Excalibur Sport V2.0; Groningen, Netherlands). During the test, VO_2_, carbon dioxide production, and minute ventilation were measured breath-by-breath by means of a validated (Rosdahl et al., 2013) metabolic cart (Moxus Modular VO_2_ System, AEI Technologies, Pittsburgh, PA). The exercise test increments were designed to exhaust the subject within 10–15 min. The test was a continuous protocol with a starting power set at 50 W for 5 min and then each stage consisted of a 1-min exercise period and increased by 20 W.

##### 2.2.1.2 Force-Velocity Test (FVT) and Wingate familiarization

After a 5 min warm-up, this test consisted of the repetition of short, maximal, cycle sprints (Lode Excalibur Sport V2.0; Groningen, Netherlands), using different braking forces. Each sprint was completed in 5 s, corresponding to the time it takes for a healthy motivated participant to attain their maximal velocity after the starting signal. In random order, participants started the test against the following three external resistances: 0.3, 0.6, or 0.8 N∙kg^−1^ of body mass. After at least a 5-min rest period between each sprint, participants were told to remain on the saddle and to perform the next sprint.

These sprints allowed us to familiarize the participants with the cycle ergometer and to calculate the optimal starting velocity to be used during sessions 2A and 2B. The velocity, torque, and power values (averaged per half-pedal revolution) recorded during the acceleration phase of the three sprints were used to draw the individual force-velocity and power-velocity relationships (Arsac et al., 1996). The optimal values of friction force and velocity at which the highest power was reached were determined from these relationships. Finally, all participants performed a single Wingate test to be familiar with this specific test. The single Wingate was performed in the same conditions defined for sessions 2A and 2B.

#### 2.2.2 Sessions 2A and 2B

##### 2.2.2.1 Exercise testing

An overview of sessions 2A and 2B is provided in Figure 1B. Participants performed a warm-up that began with 8 min against an external power of 150 W and 2 min at 260 W. This was followed by 2 min of rest before performing three 10-s efforts at 300, 400, and 500 W, each separated by 90 s of rest. Participants then rested in the sitting position for 5 min before performing the main exercise test session that consisted of 3 × 30-s Wingate tests, each separated by 20 min of rest. We reproduced a typical cycling training session used by world-class athletes at the French Sports Institute (Thomas et al., 2021). The long recovery periods used in this protocol maximise the metabolic disturbances to exercise (Hanon et al., 2012). For each all-out exercise bout, the pace was not controlled, meaning that participants were required to complete each 30 s effort with a maximal effort from the beginning. The resistance was set as 7.5% of the participant’s body mass, according to previous study protocols (Gibala et al., 2006). Participants were informed of the time elapsed after 15 and 20 s of exercise and then received a 5 s countdown until the end. The mean power over 30 s for each sprint (P_mean_), maximal power (P_max_), and instantaneous end power (P_end_) were determined. The fatigue index (FI) of the all-out test was calculated as: [(P_max_ – P_end_)/P_max_] × 100.

##### 2.2.2.2 Supplementation

Ninety minutes before the start of the exercise, participants ingested either 0.3 g∙kg^−1^ of sodium bicarbonate (BIC) or 0.2 g∙kg^−1^ of calcium carbonate (PLA) contained within gelatine capsules. Ingestion took place within 20 min and with water *ad libitum* (Edge et al., 2006). For both conditions, participants were required to drink the same quantity of water. Treatment was assigned in a counterbalanced, randomised, double-blind manner. The placebo was chosen according to previous studies (Hollidge-Horvat et al., 2000; Carr et al., 2013; Thomas et al., 2016).

##### 2.2.2.3 Rating of perceived exertion (RPE)

Immediately after each 30 s effort, and before getting off the bike and sitting down for the 20-min rest period, participants provided a rating of perceived exertion (RPE) using two scales. The first was a 15-point (6–20) scale estimating cardio-respiratory exertion, where a rating of 6 reflects exercise that is very, very easy and a rating of 20 reflects maximum exertion. The second scale, the Borg category-ratio scale (CR10), was an 11-point (0–10) scale estimating muscular pain, where 0 reflects no pain, and 10 reflects maximum pain (Borg and Kaijser, 2006).

### 2.3 Plasma analyses

Capillary blood samples (85 μL) were taken from a hyperemic fingertip at rest, immediately post-warm-up, and at 3, 5, and 9 min after each Wingate test. The samples were analysed immediately. Plasma lactate ([La^−^]) and glucose concentrations were measured using an automated lactate analyser (2300 STAT Plus, YSI Inc., Yellow Springs, OH), and pH, bicarbonate concentration ([HCO_3_^−^]), sodium concentration ([Na^+^]), and calcium concentration ([Ca^2+^]) were measured using an automated blood gas analyser (Rapidpoint 405, Siemens Healthcare, Melbourne, Australia).

### 2.4 Skeletal muscle biopsies

During both sessions 2A and 2B, four muscle biopsies were performed by an experienced medical practitioner at rest (Pre), within 10 s following the completion of the final 30 s effort (Post), and both 6 h and 24 h following exercise while at rest. Between the first biopsy and the first blood sample, all participants had a standardised breakfast and either the PLA or BIC supplementation.

On the day of Sessions 2A and 2B, two incisions were made under local anesthesia (5 mL, 1% Xylocaine) into the skin, surrounding fascia, and epimysium of the *vastus lateralis* muscle (halfway between the knee and the hip). One incision was used immediately for the resting biopsy, and the other was closed with a Steri-Strip and was used immediately after the third sprint. The biopsies at 6 h and 24 h were taken approximately 1 cm from the previous biopsy site. The muscle was taken from the *vastus lateralis* muscle of each participant using the 5-mm Bergström needle technique modified with suction (Evans et al., 1982). Muscle samples were blotted free of any visual blood on filter paper. A portion of the rest and 24 h muscle samples were immediately placed in BIOPS for measurement of mitochondrial respiration. Other portions of the muscle samples were immediately snap-frozen (less than 30 s between sampling and freezing) in liquid nitrogen and stored at −80°C until subsequent analysis. The biopsies in session 2B were taken from the opposite leg to the one used in session 2A, in order not to find connective tissue linked to the scars of the previous biopsies.

### 2.5 Mitochondrial respiration analysis

#### 2.5.1 Preparation of permeabilized skeletal muscle fibres

A 10–20 mg fresh muscle sample was placed in ice-cold BIOPS, a biopsy-preserving solution containing (in mM) 2.77 CaK_2_-EGTA, 7.23 K_2_-EGTA, 5.77 Na_2_-ATP, 6.56 MgCl_2_, 20 taurine, 50 2-(N-morpholino) ethanesulfonic acid (Mes), 15 Na_2_-phosphocreatine, 20 imidazole and 0.5 DTT adjusted to pH 7.1 (Pesta and Gnaiger, 2012). Samples were then transferred to a 6-well plate kept on ice, where the fibres were mechanically separated using pointed forceps. Fibres were subsequently permeabilised by gentle agitation for 30 min at 4 °C in BIOPS containing 50 μg∙mL^−1^ of saponin. Samples were then washed 3 times for 7 min at 4 °C by gentle agitation in MiR05, a respiration medium containing (in mM, unless specified) 0.5 EGTA, 3 MgCl_2_, 60 K-lactobionate, 20 taurine, 10 KH_2_PO_4_, 20 Hepes, 110 sucrose, and 1 g∙L^−1^ BSA essentially fatty acid-free adjusted to pH 7.1 at 37 °C (Pesta and Gnaiger, 2012). This method selectively permeabilized the cellular membrane leaving the mitochondria intact and allowing for *in situ* measurements of mitochondrial respiration.

#### 2.5.2 High-resolution respirometry

After washing, 3–4 mg wet weight of muscle fibres were assayed in duplicate in a high-resolution respirometer (Oxygraph-2k, Oroboros Instruments, Innsbruck, Austria) containing 2 mL of MiR05. Mitochondrial respiration was measured at 37 °C. Oxygen concentration (nM∙mL^−1^) and oxygen flux (pM∙s^−1^∙mg^−1^) were recorded using DatLab software (Oroboros Instruments, Innsbruck, Austria), and instrumental background oxygen flux, accounting for sensor oxygen consumption and oxygen diffusion between the medium and the chamber boundaries, was corrected online. Re-oxygenation by direct syringe injection of O_2_ in the chamber was necessary to maintain O_2_ levels between 275 and 450 nM∙mL^−1^ and to avoid potential oxygen diffusion limitation.

#### 2.5.3 Mitochondrial respiration protocol

A substrate-uncoupler-inhibitor titration (SUIT) protocol was used and the SUIT sequence, with final chamber concentration in brackets, was as follows: pyruvate (2 mM) and malate (5.1 mM) in the absence of adenylates were added for the measurement of LEAK respiration (L) with electron entry through Complex I (CI) (CI)L. ADP (5.1 mM) was then added for the measurement of maximal oxidative phosphorylation (OXPHOS) capacity (P) with electron input through CI (CI)D5.1 followed by the addition of succinate (10 mM) for measurement of P with simultaneous electron supply through CI + Complex II (CII) combined (CI + II)Max. This respiration state provides convergent electron input to the Q-junction through CI (NADH provided by malate/pyruvate) and CII (flavin adenine dinucleotide reduced (FADH_2_) provided by succinate) and supports maximal mitochondrial respiration by reconstruction of the tricarboxylic acid cycle function (Pesta and Gnaiger, 2012). Cytochrome c (10 μM) was then added to test for outer mitochondrial membrane integrity; an exclusion criterion was set such that if a chamber showed an increase in O2 flux >6% after the addition of cytochrome c, it was discarded (Kuang et al., 2022). This was followed by a series of stepwise carbonyl cyanide 4-(trifluoromethoxy) phenylhydrazone titrations (FCCP, 0.75–1.5 μM) for measurement of electron transport system (ETS) capacity (E) with convergent electron input through CI + II (CI + II)ETS. Rotenone (0.5 μM), an inhibitor of CI, was then added to obtain a measurement of E with electron input through CII (CIIE). This was followed by the addition of antimycin A (2.5 μM), an inhibitor of Complex III (CIII), to obtain a measurement of residual oxygen consumption capacity (ROX). ROX was subtracted from all other measurements to account for oxidative side reactions. Ascorbate (2 mM) and N,N,N′,N′-tetramethyl-p-phenylenediamine (TMPD, 0.5 mM), artificial electron donors for Complex IV (CIV), followed by sodium azide (>200 mM), an inhibitor of CIV to account for auto-oxidation of ascorbate + TMPD, were then added to measure CIV respiration in the non-coupled state (CIVE). Respiratory flux control ratios (FCR) were calculated.

In brief, the phosphorylation control ratio (PCR) is the quotient of (CI + II)_Max_ over (CI + II)_ETS_; the coupling control ratio is the quotient of (CI)_L_ over (CI)_D5.1_ and is equivalent to the inverse respiratory control ratio (inv-RCR); the substrate control ratio (SCR) is the quotient of (CI)_D5.1_ over (CI + II)_Max_.

### 2.6 Sample preparation for western blotting

Freeze-dried muscle samples were dissected free of visible blood, fat, and connective tissue and then thoroughly homogenised using a loose-fitting Dunce (Teflon-glass) homogeniser in lysate buffer [Sucrose 210 mM, Hepes 30 mM, EGTA 2 mM, NaCl 40 mM, EDTA 6 mM pH 7.4] complemented with protease and phosphatase inhibitors (Sigma) and centrifuged for 10 min at 10,000 g at 4°C. The protein concentration of the supernatant was determined with the BCA protein assay kit (Pierce, Interchim, Montluçon, France), and prior to freezing at −80°C, aliquots of supernatant were performed in order to be used for Western Blotting and to determine enzyme activity (7 µL) and oxidative stress markers (50 µL). There was no significant difference in the protein concentration of muscle samples isolated from the PLA and BIC conditions (5.1 ± 0.7 vs. 5.2 ± 0.8 g∙μL^−1^; *p* > 0.05). Supernatants of muscle homogenates (20 µg of protein) and a pre-stained molecular mass marker (Euromedex 06P-0211, 10–250 kDa) were separated on Nupage 4%–12% BisTris gels of 1.5 mm (200 V for 60 min – NP0335BOX) with the Novex system (Invitrogen, Groningen, Netherlands) in buffer containing MOPS-SDS [1M MOPS, 1M Tris Base, 69.3 mM SDS, 20.5 mM EDTA, H2O to adjust the dilution, pH 7.7]. A large muscle specimen from a participant who later withdrew from the study was used as an internal control. This sample was loaded in each gel, and lanes were normalised to this value to reduce gel-to-gel variability. Proteins were transferred from the gels to nitrocellulose membranes using a semi-dry method (101 mA for 75 min), and membranes were then incubated on a shaker for 90 min in a blocking buffer at room temperature (Odyssey^®^ Blocking Buffer; LI-COR, Cat. #927–40000; 10X phosphate-buffered saline (PBS) [140 mM NaCl, 2.68 mM KCl, 10.14 mM Na_2_HPO_4_, 1.76 mM KH_2_PO_4_, pH 7.4] containing 0.1% sodium azide; Euromedex, Cat. #OORA00260). The membranes were incubated overnight at 4°C with primary antibodies, which were each diluted at 1:1,000 in blocking buffer, except for α-tubulin 1:2,000. We assayed MCT1 and MCT4 [personal antibodies (Thomas et al., 2005)], CD147 (sc-9757 Santa Cruz), NHE1 (MAB3140 Millipore), NBC (AB3212 Millipore), CAII (sc-25596 Santa Cruz), peroxisome proliferator-activated receptor gamma coactivator 1-α (PGC-1α: 4,259 Cell Signaling Technology), cytochrome c oxidase subunit 4 (COX IV: 4,844 Cell Signaling Technology). This was followed by three 5-min washes in PBS-Tween buffer (PBS 1X + 0.25% Tween 20), and one 5 min wash in PBS 1X buffer. Then, membranes were incubated for 45 min with the secondary antibody in a blocking buffer. Membranes were washed again as previously described, and protein expression was detected by enhanced immunofluorescence (Odyssey Infrared Imaging System LI-COR Biosciences, ScienceTec, Courtabœuf, France). ɑ-tubulin (T6074 Sigma-Aldrich, 1:2,000) was used as a loading control to ensure correct loading of protein content in each lane. Protein band densities were calculated from the sum of the peak intensities using the ImageJ software (http://rsbweb.nih.gov/ij/index.html). Each band density is reported as related to α-tubulin as the loading control and expressed with the internal standard. Levels of protein expression in each sample were corrected using the respective quantified levels of the corresponding loading control analysed on the same membrane.

### 2.7 Muscle enzyme analysis

Citrate synthase (CS) activity was measured with 2 µL of supernatants of muscle homogenate from western blotting preparation in 100 mmol/L Tris HCl (pH = 8.0) buffer with 0.5 mmol/L oxaloacetate, 0.3 mmol/L acetyl-CoA, 0.1 mmol/L 5,5′-dithiobis 2-nitrobenzoic acid (DTNB) as previously described (Srere, 1969). The increase in absorbance at 412 nm was followed over 3 min at 25°C due to the formation of the 5-thio-2-nitrobenzoate anion (TNB) generated from DTNB. CS activity was expressed as micromoles of TNB formed per minute per Gram of tissue.

### 2.8 Markers of oxidative stress

#### 2.8.1 Protein carbonylation

Protein carbonylation was determined according to the Oxyblot protein oxidation detection kit (kit OxyBlot S7150, Millipore). 5 µL of 12% SDS was added to each previously supernatants of muscle sample (5 µL) containing 15 µg of protein. All samples were then reduced in 2,4-Dinitrophenol (2,4-DNP) after a 15-minute incubation period in 10 µL of 2,4-Dinitrophenylhydrazine (2,4-DNPH) (“sample of interest”). At the same time, a “negative control” sample was prepared similarly, but the 2,4-DNPH was replaced by 10 µL of a standard reduction solution (Oxyblot). After 15 min at room temperature, 7.5 µL of neutralisation buffer (Oxyblot) was added to each sample with a 5% volume of 2-mercaptoethanol. Then, the “sample of interest” and the “negative control” were both run on Nupage 4%–12% BisTris gels of 1.5 mm (200 V for 60 min – NP0335BOX) and transferred on nitrocellulose membranes. Membranes were first saturated in the blocking buffer for 1 h at room temperature and then incubated with the primary antibody (Oxyblot) (1:150) overnight at 4°C in the blocking buffer. After four washes (same protocol as described for western blots), membranes were incubated for 45 min with the secondary antibody (Oxyblot) (1:300) coupled with HRP (horseradish peroxidase) at room temperature. The quantification was done using all the bands of the sample and expressed relative to the internal standard.

#### 2.8.2 Lipid peroxidation

The 4-hydroxy-2-nonenal (4-HNE: sc-130083 Santa Cruz) analysis is based on the same principle used for classic western blots, with a dilution of the primary antibody equal to 1:3,000 and 1:1,000 for the secondary antibody. The quantification was done using all the bands of the sample and expressed relative to the internal standard.

### 2.9 Statistical analyses

All data were tested for normality using the Shapiro-Wilk test and are reported as means and standard deviation (mean ± SD). No data were found to significantly deviate from a normal distribution. Data were analysed using a two-way (condition x time) repeated-measures ANOVA. Where significance was achieved, Tukey’s *post hoc* analysis was undertaken to localise the difference. Significance was set *a priori* at the level of *p* < 0.05. All analyses were performed using SigmaPlot (Systat Software, Inc., San Jose, California, United States).

## 3 Results

### 3.1 Power outputs and RPE

The results for performance during each of the 3 × 30-s Wingate s tests, each separated by 20 min of rest, are summarised in Table 1. There were no significant time effects for P_max_, P_mean_, or FI (*p* > 0.05). There were also no significant condition or interaction effects for mechanical performance variables. In addition, there were no significant time or condition effects for both RPE scales (Borg = PLA: 15.6 ± 0.1 vs. BIC: 16.0 ± 0.4; *p* > 0.05; CR10 = PLA: 7.8 ± 0.1 vs. BIC: 7.2 ± 0.02; *p* > 0.05).

**TABLE 1:** Power parameters for the 3 × 30-s Wingate Test (*n* = 8, Mean ± SD). Fatigue Index = [(P_max_ – P_end_)/P_max_] × 100.

	Wingate number	Maximal power (W)	Mean power (W)	Fatigue index (%)
BIC	1st	876 ± 211	660 ± 161	49.3 ± 6.2
	2nd	767 ± 201	620 ± 165	39.8 ± 11.1
	3rd	856 ± 293	633 ± 166	48.1 ± 13.5
	***Mean ± SD***	***833 ± 50***	***637 ± 3.0***	***45.7 ± 3.7***
PLA	1st	885 ± 320	664 ± 159	50.1 ± 12.9
	2nd	825 ± 237	645 ± 129	46.8 ± 9.9
	3rd	879 ± 297	657 ± 146	55.5 ± 7.3
	***Mean ± SD***	***863 ± 43***	***655 ±* 15**	***50.8 ± 2.8***
p-value (condition)		0.70	0.70	0.26
p-value (time)		0.60	0.86	0.09
p-value (interaction)		0.98	0.98	0.44

The bold values represent the Mean of the three Wingate Test.

### 3.2 Blood plasma parameters

**FIGURE 2:**
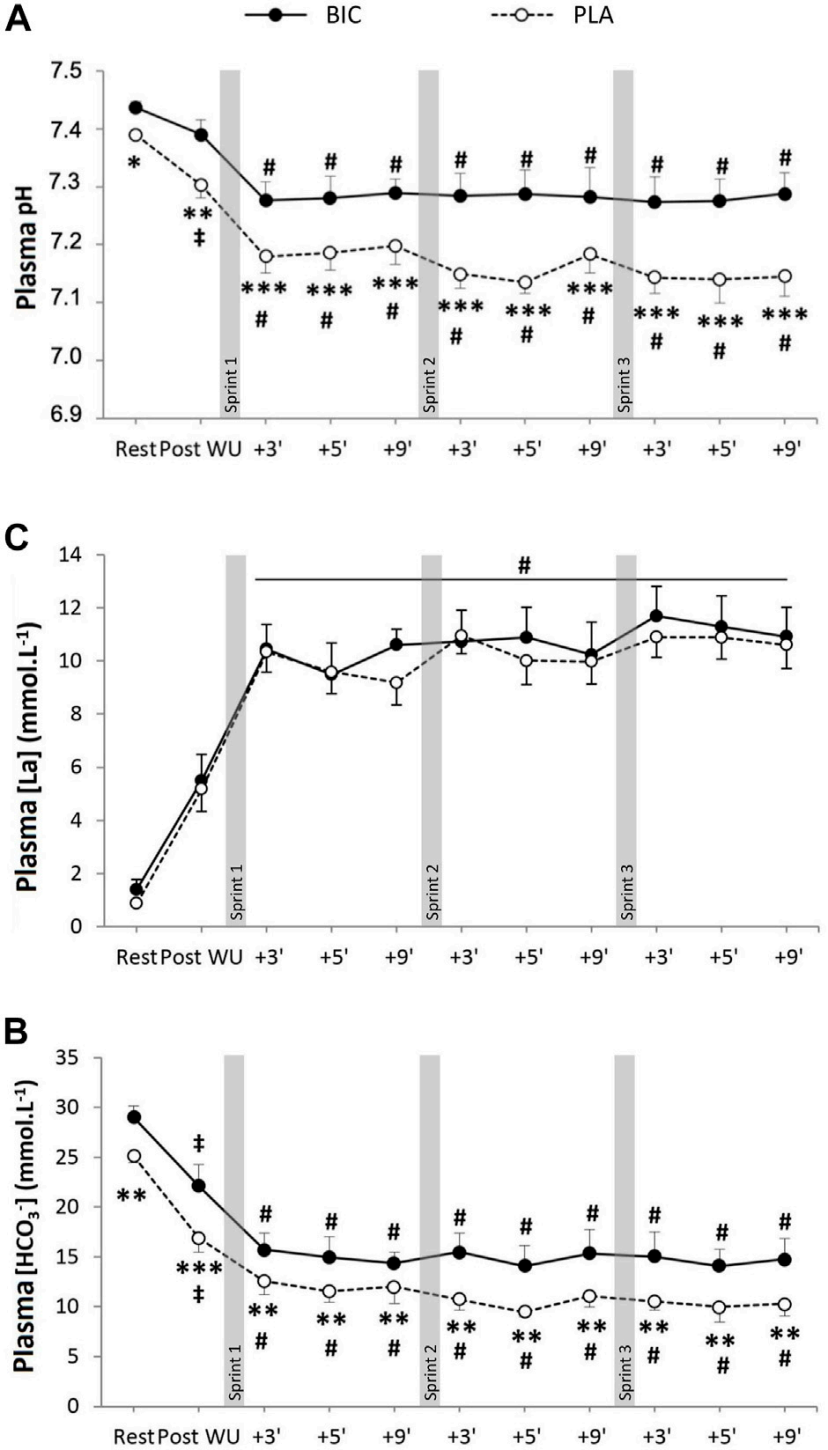
**(A)** Blood pH, **(B)** bicarbonate concentration ([HCO3^−^]), and **(C)** lactate concentration ([La^−^]) during the repeated sprints after either BIC (solid line) or PLA (dashed line) supplementation (*n* = 8). *, **, *** denote significant differences from PLA (*p* < 0.05, *p* < 0.01 and *p* < 0.001, respectively); # denotes significant difference from post warm-up (*p* < 0.001); ‡denotes significant differences from rest (*p* < 0.05).

There was a significant main effect of time for both plasma pH and [HCO_3_^−^], with both parameters significantly lower post-exercise compared to rest (Figure 2). There were also significant condition and interaction effects (time x condition) for both parameters, with both plasma pH and [HCO_3_^−^] significantly lower in PLA *versus* BIC at all time points. There was a significant main effect for time but not condition for [La^−^] ([La^−^]_max_: PLA: 10.9 ± 0.7 vs. BIC: 11.7 ± 1.1 mM∙L^−1^; *p* > 0.05). No significant effect of time or condition was observed for either glucose concentration or [Na^+^] and [Ca^2+^] (*p* < 0.05; data not shown).

### 3.3 Lactate and pH regulation

#### 3.3.1 MCT1 and MCT4

**FIGURE 3:**
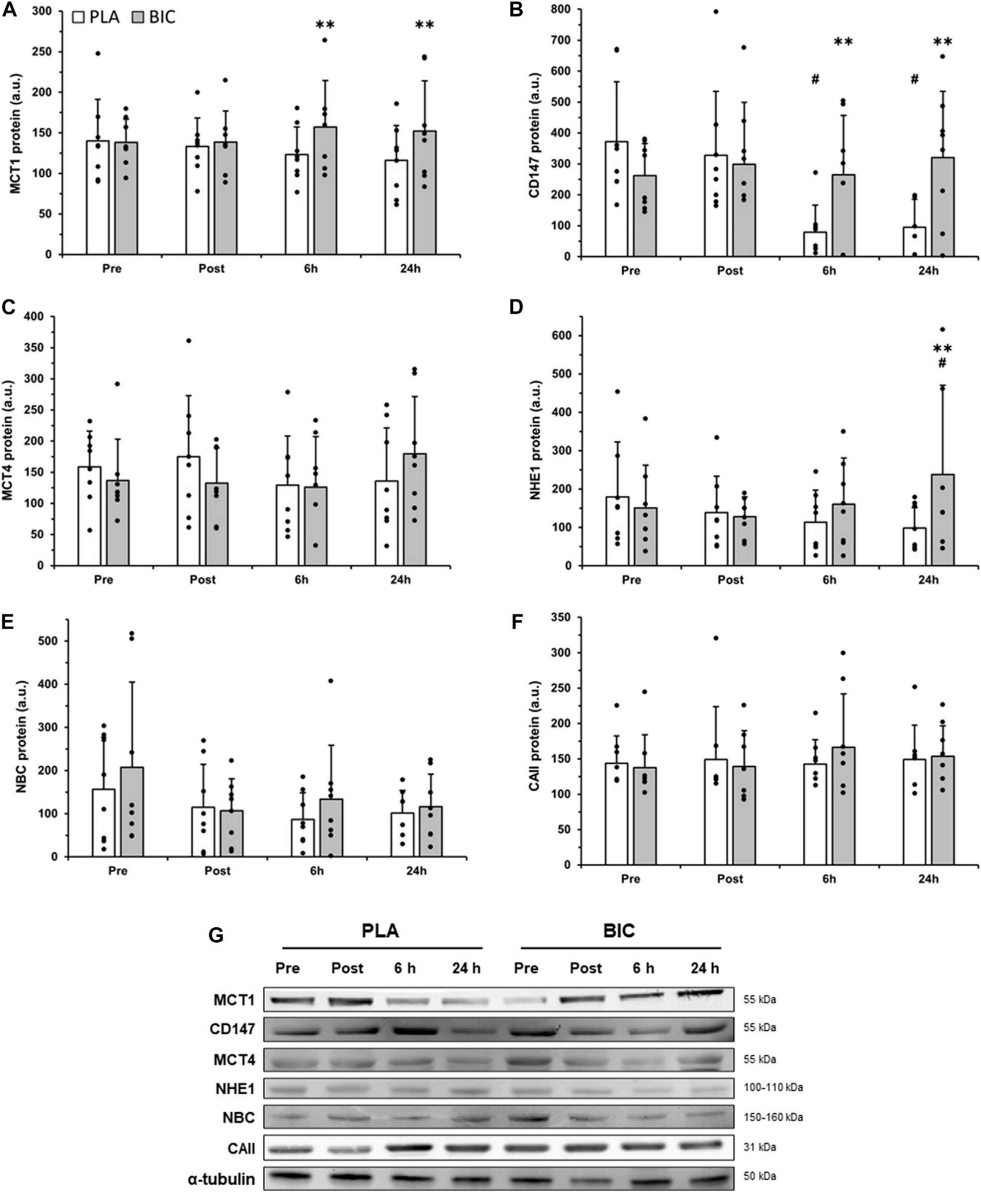
Comparison of **(A)** MCT1, **(B)** CD147, **(C)** MCT4, **(D)** NHE1, **(E)** NBC, and **(F)** CAII protein abundance in the *vastus lateralis* between the PLA (white bars) and BIC (grey bars) conditions. Values are normalized to the α-tubulin loading control and expressed in arbitrary units. All values are mean ± SD (*n* = 8). **(G)** Representative western blots for each protein. α-tubulin served as a loading control, and a biopsy from a single participant who did not complete the intervention served as a positive control. **Denotes significant differences from PLA (*p* < 0.01), # denotes significant differences from rest (*p* < 0.01).

There was a significant interaction effect for MCT1 content (*p* < 0.05, Figure 3), with *post hoc* analyses revealing that MCT1 content was significantly higher in BIC compared to PLA 6 h (+21.6%) and 24 h (+23.7%) after the end of the 3 × 30-s Wingate Test, whereas there was no significant main effect for time or condition, nor interaction effects, for MCT4 content (Figure 3).

#### 3.3.2 CD147

There was a significant interaction effect for the content of the MCT1 chaperone protein CD147 (*p* < 0.05; Figure 3). In the PLA condition, CD147 content was significantly lower after both 6 h and 24 h of recovery compared to Pre (−78.8% and −74.5% respectively; *p* < 0.01). In addition, CD147 content (*p* < 0.01) was higher in BIC compared to PLA after 6 h (+70.3%) and 24 h (+70.4%) of recovery.

#### 3.3.3 NHE1

The muscle content of NHE1 remained unchanged immediately post-exercise and 6 h after the 3 × 30-s Wingate Test in both conditions, but was significantly increased 24 h after the 3 × 30-s Wingate Test in BIC. After 24 h of recovery, the relative abundance of NHE1 was 57.7% higher compared to the Pre values in the BIC condition (*p* < 0.01) and was 58.7% higher in BIC compared to PLA (*p* < 0.01; Figure 3).

#### 3.3.4 NBC and CAII

No significant differences were observed after the 3 × 30-s Wingate Test across biopsy time points and conditions (BIC vs. PLA) for NBC or CAII relative abundance (*p* > 0.05, Figure 3).

### 3.4 Mitochondrial respiration and adaptation

#### 3.4.1 Mitochondrial respiration

**FIGURE 4:**
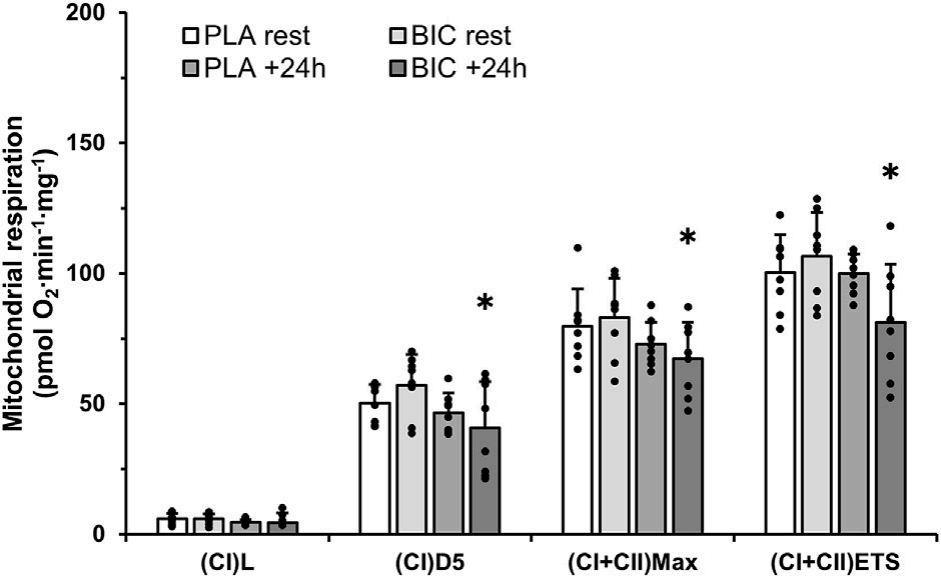
Mitochondrial respiration measurements in permeabilised muscle fibres (*vastus lateralis*), obtained from muscle biopsies taken before and 24 h following exercise in the BIC and PLA conditions (*n* = 8). Oxygen consumption values are normalised to whole tissue. All values are mean ± SD. (CI)L, leak respiration; (CI)D5, electron input through complex I; (CI + II)Max, maximal convergent electron input through complex I and II; (CI + II)ETS, Electron Transport System through complex I and II. *Denotes significant differences from rest in BIC condition (*p* < 0.05).

Mass-specific mitochondrial respiration values (results expressed as pM O_2_∙s^−1^∙mg^−1^ of tissue) are presented in Figure 4. There were no changes in mitochondrial respiration within the PLA condition. However, we reported decreases in (CI)D5.1 (−28%), (CI + II)Max (−17%), and (CI + II)ETS (−23%) (all *p* < 0.05), in the BIC condition 24 h upon the termination of exercise (Figure 4). The data presented in this study showed no effect of the addition of cytochrome c as a control for outer mitochondrial membrane integrity. There were no significant differences in any of the FCRs measured (all *p* > 0.05; Table 2).

**TABLE 2:** Respiratory flux control ratios.

FCR		PCR	Inv-RCR	SCR
**BIC**	Pre	0.78 ± 0.04	0.10 ± 0.03	0.69 ± 0.04
	24 h	0.84 ± 0.06	0.10 ± 0.11	0.58 ± 0.16
**PLA**	Pre	0.80 ± 0.05	0.12 ± 0.03	0.64 ± 0.09
	24 h	0.73 ± 0.08	0.10 ± 0.03	0.64 ± 0.06

PCR, phosphorylation control ratio (CI + II)_Max_/(CI + II)_ETS_;

Inv-RCR, inverse of respiratory control ratio (CI)_L_/(CI)_D5.1_;

SCR, substrate control ratio: (CI)_D5.1_/(CI + II)_Max_.

FCRs were calculated from mass-specific respiration measurements in permeabilized muscle fibers (*vastus lateralis*), obtained from muscle biopsies taken before 3 × 30-s Wingate Test and after 24 h of recovery after either BIC (sodium bicarbonate) or PLA (placebo) supplementation (*n* = 8, Mean ± SD).

#### 3.4.2 COX IV, PGC-1α and CS activity

**FIGURE 5:**
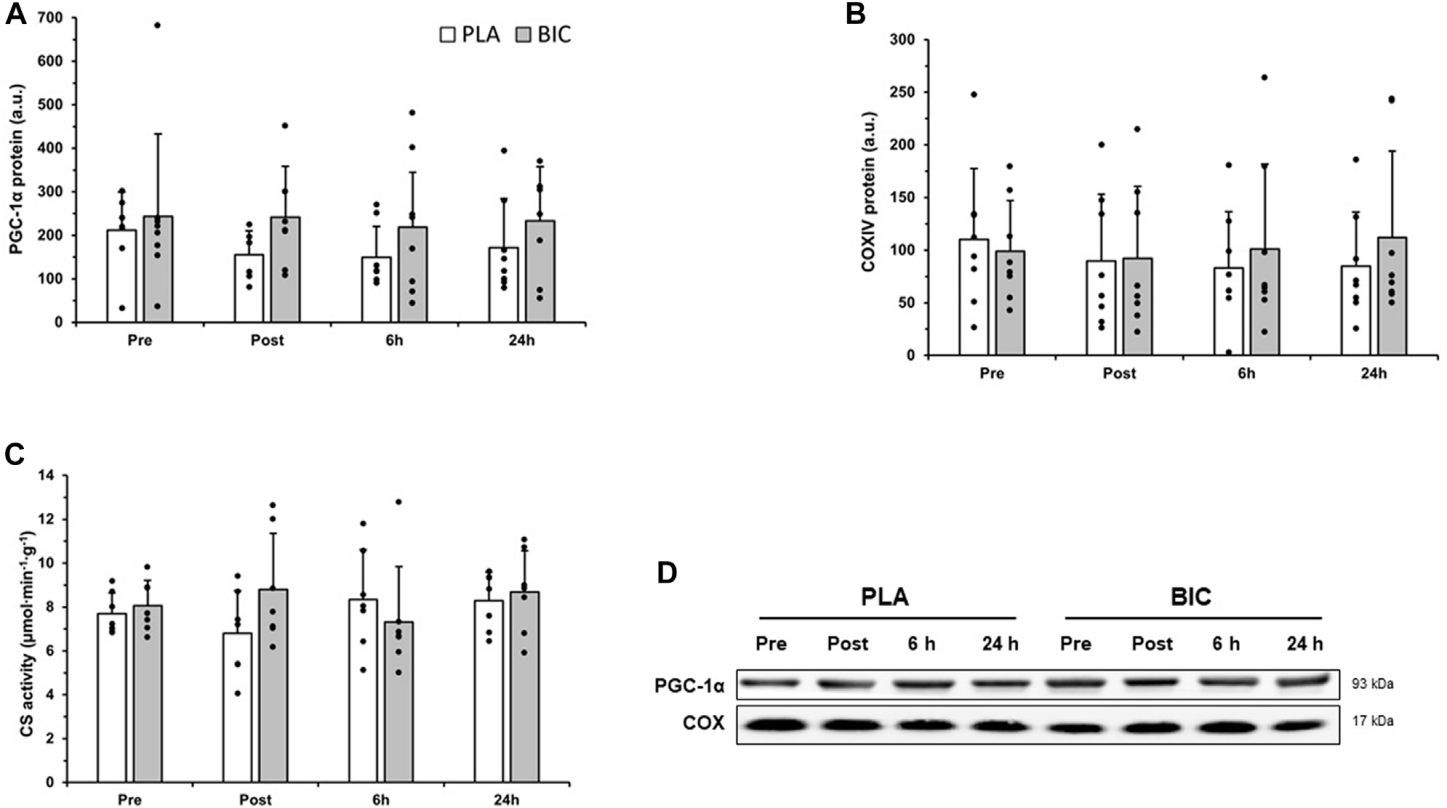
Comparison of **(A)** PGC-1α, and **(B)** COXIV protein abundance in the *vastus lateralis* between the PLA (white bars) and BIC (grey bars) conditions. Values are normalized to the α-tubulin loading control and expressed in arbitrary units. All values are mean ± SD (*n* = 8). Individual data is shown in black points. **(C)** Comparison of citrate synthase (CS) activity between the PLA (white bars) and BIC (grey bars) conditions. Values are mean ± SD (*n* = 8). **(D)** Representative western blots for each protein.

No significant differences were observed after the 3 × 30-s Wingate Test across biopsy time points and conditions (BIC vs. PLA) for COX IV or PGC-1α relative abundance, or CS activity (*p* > 0.05; Figure 5).

### 3.5 Markers of oxidative stress

**FIGURE 6:**
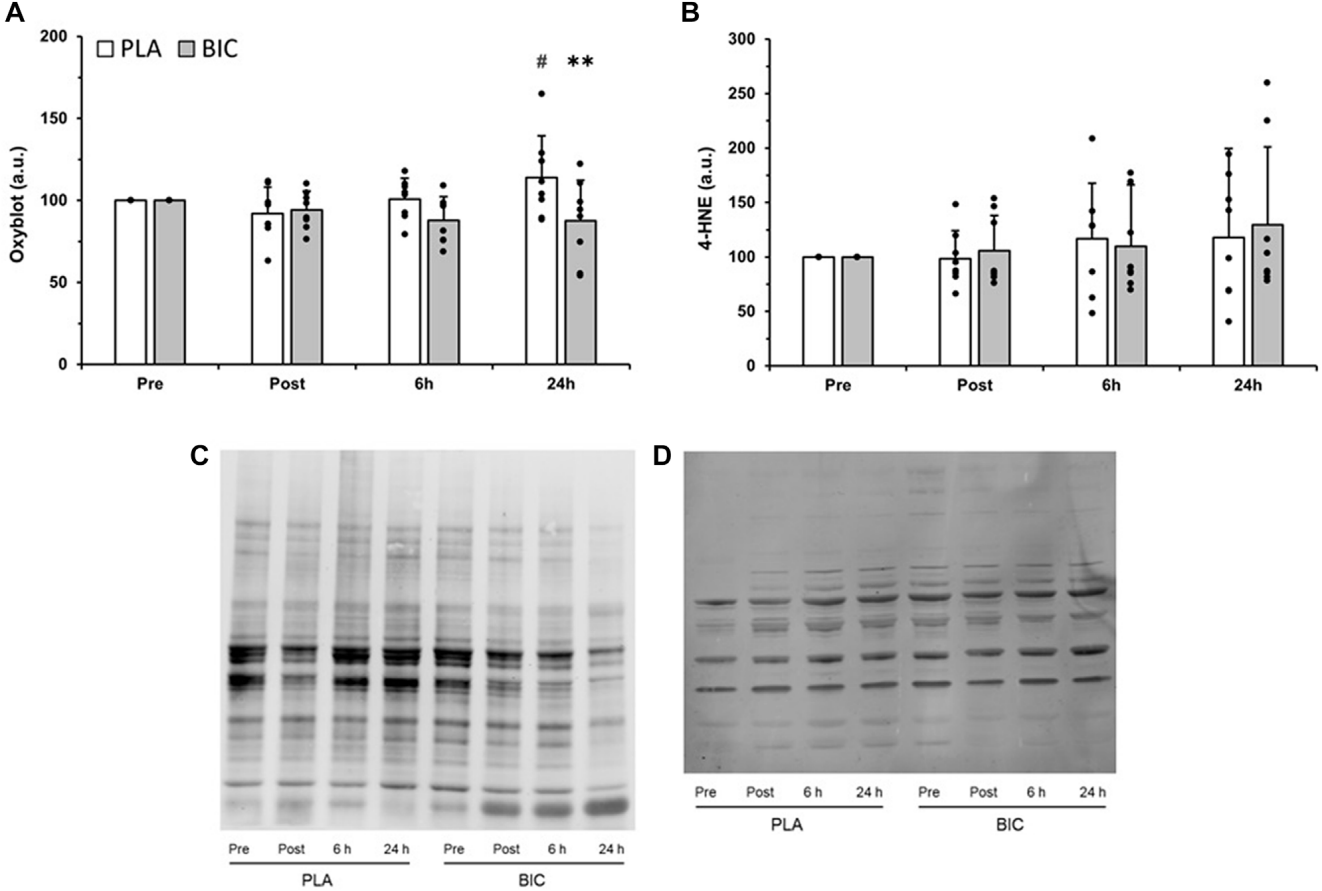
Comparison of **(A)** protein carbonylation as measured by oxyblot, and **(B)** lipid peroxidation, as measured by 4-hydoxy-2-nonenal (4-HNE), in the *vastus lateralis* between the PLA (white bars) and BIC (grey bars) conditions. All values are mean ± SD (*n* = 8). Individual data is shown in black points. **Denotes significant differences from PLA (*p* < 0.01), #denotes significant differences from rest (*p* < 0.01). Representative blots for **(C)** oxyblot and **(D)** 4-HNE are shown.

Several oxidised protein bands were detected in the *vastus lateralis* muscle of our participants, and there was a significant interaction (time x condition) for the relative abundance of total proteins oxidised (*p* < 0.05). As shown in Figure 6, oxidised protein content was 23.8% greater in the PLA condition 24 h after the end of the 3 × 30-s Wingate Test compared to immediately post-exercise (*p* < 0.01). Furthermore, values were significantly lower in the BIC condition compared to the PLA condition 24 h after the end of the 3 × 30-s Wingate Test (−30%; *p* < 0.01). There was no statistically significant effect of time or condition for lipid peroxidation (i.e., 4-HNE) (*p* > 0.05, Figure 6).

## 4 Discussion

The major novel finding from this study is that despite no difference in power, pre-exercise ingestion of NaHCO_3_ resulted in significantly greater relative protein abundance of MCT1, CD147, and NHE1 6 h and 24 h post-exercise compared with ingestion of a placebo, with no changes in MCT4, NBC, or CAII in either condition. Furthermore, a decrease in mitochondrial respiration was observed after 24 h of recovery in the BIC condition only. Compared to PLA, there was significantly lower protein carbonylation after 24 h of recovery in the BIC condition.

Participants were provided with an oral dose of sodium bicarbonate 90 min before the 3 × 30-s Wingate Test in a counterbalanced, randomised, double-blind manner. Consistent with earlier studies (Hollidge-Horvat et al., 2000; Bishop et al., 2004), sodium bicarbonate ingestion resulted in a significantly higher blood pH and [HCO_3_^−^] before, during, and after exercise, compared to the PLA condition (Figure 1; *p* < 0.05). There was, however, no significant difference between conditions for post-exercise [La], which is consistent with our previous data using identical protocols of supplementation during very high-intensity exercise (Thomas et al., 2016). There was no significant effect on changes in blood [Na^+^] or [Ca^2+^]. In both conditions, participants presented a power profile during the 3 × 30-s Wingate Test that has classically been observed in sprint athletes (Granier et al., 1995). There was a large decrease in mean power during each of the three 30 s sprints in both conditions (FI of 45%–50%), without any significant differences between conditions for perceived exertion. Importantly, no differences in the decrease in power within or between sprints were observed between the two conditions (Table 1), which is consistent with the results of other studies that have induced alkalosis before similar exercise in humans (Vanhatalo et al., 2010; Zabala et al., 2011). Consequently, any differences in protein expression or protein carbonylation between conditions could not be attributed to differences in power output.

The monocarboxylate transporters MCT1 and MCT4 are correctly targeted to the membrane by the action of their chaperone CD147 (Kirk et al., 2000; Schneiderhan et al., 2009). We found that the protein content of MCT1 and CD147 were concomitantly increased in BIC compared to PLA at 6 h and 24 h post-exercise. This is the first observation in humans consistent with a co-regulation of MCT1 and CD147 in response to contractile activity, as previously observed in diabetic rats (Nikooie et al., 2013). Although not significant, MCT4 expression also increased by 42.5% from 6 to 24 h post-exercise in BIC, compared to only a 5.3% increase in PLA (*p* > 0.05). These data suggest that NaHCO_3_ ingestion alters the molecular responses to high-intensity exercise, leading to an increase in both MCT1 and CD147 protein expression. Our results support previous findings in rats that MCT1 and MCT4 can be rapidly and substantially upregulated (∼60%) by a single session of exercise (Coles et al., 2004), but we add the novel observations that this is accompanied by their chaperone CD147 and influenced by the pre-exercise ingestion of sodium bicarbonate.

We next investigated if sodium bicarbonate affected the exercise-induced changes in other proteins involved in skeletal muscle pH regulation, such as NHE1, NBC, and CAII. Consistent with the results for MCT1, there was an increase in NHE1 24 h post-exercise in BIC. NHE1 content was not significantly altered in PLA at any measured time following the 3 × 30-s Wingate Test (Figure 3). This suggests that NHE1 upregulation does not occur rapidly after high-intensity exercise in the absence of pre-exercise alkalosis, and the repetition of several exercise sessions may be needed to observe an increase in NHE1 protein expression. This is supported by increases in NHE1 protein expression after 4 weeks but not after 2 weeks of training (Juel et al., 2004; McGinley and Bishop, 2016).

The 3 × 30-s Wingate Test did not affect the content of NBC during the first 24 h of recovery in both conditions. As NBC content was investigated in the *vastus lateralis* in our study, and NBC content has previously been shown to be negatively correlated with the percentage of type I muscle fibres (Kristensen et al., 2004), it is possible that an upregulation is more likely to be observed in muscle consisting predominantly of fast-twitch fibres. Furthermore, CAII, a protein that has been found to significantly enhance both NBC (Schueler et al., 2011) and MCT1 (Becker and Deitmer, 2008; Becker et al., 2011; Stridh et al., 2012) activities, was not upregulated by the 3 × 30-s Wingate Test in either condition. Together, these results indicate that, despite interactions between MCT1, NBC, and CAII, these proteins are associated with different regulation kinetics during the first 24 h of recovery following three all-out sprints.

As metabolic acidosis has been reported to influence oxidative capacity (Jubrias et al., 2003), we also sought to investigate whether mitochondrial respiration would be affected by the pre-exercise ingestion of sodium bicarbonate. In the present study, the significantly higher blood pH in BIC was accompanied by a significant decrease in mitochondrial respiration after 24 h. This is in line with the contrasting effect of physiological decreases in pH, which induce an increase in mitochondrial respiration in cells (Khacho et al., 2014), and in *ex vivo* muscle (Tonkonogi and Sahlin, 1999). The acute changes in mitochondrial respiration observed in the present training do not appear to be attributable to changes in mitochondrial content, as there were no post-exercise changes in common markers of mitochondrial content (e.g., CS activity, and the relative protein abundance of COX IV and PGC-1α). These findings are in agreement with previous results in whole muscle (Little et al., 2011; Percival et al., 2015). We also add that there was a dissociation between post-exercise changes in mitochondrial protein abundance and mitochondrial respiration, which has previously been reported after a period of exercise training (Vincent et al., 2015; Granata et al., 2016; 2021). Interestingly, we have previously reported that chronic bicarbonate supplementation throughout an interval training program in rats resulted in improvements in mitochondrial respiration (Bishop et al., 2010). It is possible that the transient loss of mitochondrial function following sprint interval exercise with BIC may stimulate subsequent adaptations that contribute to greater improvements in mitochondrial respiration following chronic training.

Since the production of reactive oxygen species is increased after a single session of high-intensity exercise (Radak et al., 2013), and regulates signaling pathways and protein expression, we also investigated markers of oxidative stress. Indeed, one possible candidate to help to explain the greater MCT1, CD147, and NHE1 protein content in BIC compared to PLA is a decrease in oxidative stress after 24 h of recovery (Liu et al., 2012). While we did not observe changes in lipid peroxidation, muscle protein carbonylation was increased 24 h after the end of exercise in the PLA condition (Figure 5). In contrast, exercise-induced changes in markers of protein carbonylation were significantly reduced by pre-exercise alkalosis, consistent with reports that NaHCO_3_ treatment can suppress both hydrogen peroxide accumulation and superoxide dismutase activity (Liu et al., 2012), and that hydrogen peroxide can induce carbonylation of proteins (Costa et al., 2002). As protein carbonylation is an irreversible post-translational modification that often leads to the loss of protein function and could affect protein expression (Radak et al., 2013), this may have contributed to the reduction in MCT1, CD147, and NHE1 protein content in the PLA condition compared to the BIC condition. However, it is important to note that significant differences in protein carbonylation between conditions were only observed at 24 h, and not 6 h, even though significant differences in CD147 and MCT1 content were already observed at 6 h, suggesting other mechanisms are likely to contribute to the differences in CD147 and MCT1 content between conditions.

In summary, this study revealed that metabolic alkalosis during sprint-interval exercise increases the protein expression of MCT1, CD147 and NHE1, decreases mitochondrial respiration, and decreases reactive oxygen species in the post-exercise recovery period. These changes could not be attributed to increases in work capacity or [La^−^]. These findings are consistent with evidence that pre-exercise sodium bicarbonate promotes greater skeletal muscle adaptations to regulators of skeletal muscle lactate concentration in rats (Thomas et al., 2007). Finally, we reveal an unexpected role for the pre-exercise ingestion of sodium bicarbonate to decrease mitochondrial respiration during the recovery from very high-intensity exercise. Given that chronic supplementation of sodium bicarbonate enhances mitochondrial respiration following long-term training (Bishop et al., 2010), it is possible that this transient decrease in mitochondrial respiratory function is involved in inducing the supercompensation necessary for long-term improvements in mitochondrial respiration. This finding has direct implications for athletes using sodium bicarbonate supplements during the competition season. Together, these results serve as a direction for future research to evaluate how pH interacts with exercise to mediate adaptation.

## Data Availability

The raw data supporting the conclusion of this article will be made available by the authors, without undue reservation.
